# PERFORM: A System for Monitoring, Assessment and Management of Patients with Parkinson's Disease

**DOI:** 10.3390/s141121329

**Published:** 2014-11-11

**Authors:** Alexandros T. Tzallas, Markos G. Tsipouras, Georgios Rigas, Dimitrios G. Tsalikakis, Evaggelos C. Karvounis, Maria Chondrogiorgi, Fotis Psomadellis, Jorge Cancela, Matteo Pastorino, María Teresa Arredondo Waldmeyer, Spiros Konitsiotis, Dimitrios I. Fotiadis

**Affiliations:** 1. Unit of Medical Technology and Intelligent Information Systems, Dept. of Materials Science and Engineering, University of Ioannina, P.O. Box 1186, GR 45110 Ioannina, Greece; E-Mails: atzallas@cc.uoi.gr (A.T.T.); markos@cs.uoi.gr (M.G.T.); rigas@cs.uoi.gr (G.R.); ekarvuni@cc.uoi.gr (E.C.K.); 2. Dept. of Informatics and Telecommunication Engineering, University of Western Macedonia, GR 50100 Kozani, Greece; E-Mail: dtsalikakis@uowm.gr; 3. Dept. of Neurology, Medical School, University of Ioannina, GR 45110 Ioannina, Greece; E-Mails: mariachond@gmail.com (M.C.); skonitso@uoi.gr (S.K.); 4. ANCO S.A. Research and Development Division, 44, Syngrou Avenues, Athens 11742, Greece; E-Mail: fpso94@otenet.gr; 5. Life Supporting Technologies, Universidad Politécnica de Madrid, Madrid 28040, Spain; E-Mails: mpastorino@lst.tfo.upm.es (M.P.); jcancela@lst.tfo.upm.es (J.C.); mta@lst.tfo.upm.es (M.T.A.W.); 6. Campus de Excelencia Internacional Campus Moncloa, Universidad Complutense de Madrid - Universidad Politecnica de Madrid, Madrid 28040, Spain

**Keywords:** Parkinson's disease (PD), motor symptoms, remote monitoring, wearable devices

## Abstract

In this paper, we describe the PERFORM system for the continuous remote monitoring and management of Parkinson's disease (PD) patients. The PERFORM system is an intelligent closed-loop system that seamlessly integrates a wide range of wearable sensors constantly monitoring several motor signals of the PD patients. Data acquired are pre-processed by advanced knowledge processing methods, integrated by fusion algorithms to allow health professionals to remotely monitor the overall status of the patients, adjust medication schedules and personalize treatment. The information collected by the sensors (accelerometers and gyroscopes) is processed by several classifiers. As a result, it is possible to evaluate and quantify the PD motor symptoms related to end of dose deterioration (tremor, bradykinesia, freezing of gait (FoG)) as well as those related to over-dose concentration (Levodopa-induced dyskinesia (LID)). Based on this information, together with information derived from tests performed with a virtual reality glove and information about the medication and food intake, a patient specific profile can be built. In addition, the patient specific profile with his evaluation during the last week and last month, is compared to understand whether his status is stable, improving or worsening. Based on that, the system analyses whether a medication change is needed—always under medical supervision—and in this case, information about the medication change proposal is sent to the patient. The performance of the system has been evaluated in real life conditions, the accuracy and acceptability of the system by the PD patients and healthcare professionals has been tested, and a comparison with the standard routine clinical evaluation done by the PD patients' physician has been carried out. The PERFORM system is used by the PD patients and in a simple and safe non-invasive way for long-term record of their motor status, thus offering to the clinician a precise, long-term and objective view of patient's motor status and drug/food intake. Thus, with the PERFORM system the clinician can remotely receive precise information for the PD patient's status on previous days and define the optimal therapeutical treatment.

## 1. Introduction

Neurodegenerative diseases are characterized by the progressive loss of neurons in the central nervous system. The most common disorders are Alzheimer's disease and Parkinson's disease (PD) [1]. The risk to develop those devastating diseases increases sharply with age: PD affects 1% of the population over 65 years of age, rising to 2% for those over 80 years [2]. With an increasingly ageing population, neurodegenerative diseases will assume greater importance. The cases for PD are expected to double worldwide by the year 2020. Proper medical care of these patients is getting increasingly complex and expensive. Lengthy hospital stays for monitoring and adjustment of the patients' treatment and the problems related with it, contribute to cost increase and morbidity due to the hospitalization itself.

In the current medical practice, assessment of PD motor disabilities is based on neurological examination during patient's visits to the clinic and home diaries that the patient or the caregiver keeps. However, the short-time examination may not reveal important information to the neurologist while data from the daily diaries are highly subjective since they rely on the patient's memory and perception of his own symptoms. In addition, most of the patients may not be aware of mild symptoms or not be able to identify early “wearing off”, while they may unconsciously exaggerate or attenuate symptoms' severity. To address these problems and to find more objective assessments, several rating scales have been used, with the Unified Parkinson's Disease Rating Scale (UPDRS) being the most widely used [3]. UPDRS is a rating scale that quantifies selected symptoms and signs of PD in a 5-point scoring system. Unfortunately, UPDRS presents intra and inter-observer inconsistencies, while its use is limited to the patient's visits to the hospital. Also, the pattern and severity of PD symptoms may vary considerably during the day, while clinical rating scales only provide moment-to-moment assessments; and finally, measurements of motor functions made in the clinic may not accurately reflect the actual motor disabilities experienced by the patients in their daily life. In addition to rating scales, akinesia and gait are sometimes evaluated by means of other tests (timed motor performance test, Purdue pegboard test, pronation-supination test, finger dexterity, *etc.*). Also, objective methods have been suggested to quantify rigidity [4]. While these methods are quantitative, again they only provide information limited to the clinic settings. To overcome these limitations ambulatory monitoring of PD motor symptoms methods have been presented in the literature.

In this paper, an intelligent system for monitoring neurodegenerative disease evolution through the employment of a wide range of wearable accelerometers and gyroscopes has been developed. This system has been evaluated using short-term and long-term recordings from several PD patients. The system provides to health professionals a powerful decision support tool, and to patients a ubiquitous robust health evaluation and monitoring system, which feedback them with the appropriate medical information when necessary. The main contributions of the system are summarized below:

- A multi-parametric wearable monitoring system to recognize health status and detect the cardinal motor symptoms of PD patients.
- An intelligent closed-loop mechanism, which inform the patient about the appropriate actions to reduce diseases signs and progression.
- A remote monitoring and health care support with intelligent decision tools that assist general practitioners and specialized physicians to design better and personalized patient-specific treatment regime when and where needed.

## 2. Related Work

A limited number of movement analysis systems have been described for the ambulatory measurement of the various aspects of movement disorders in PD. Electromyography (EMG) has been used for a long time to study tremor in PD, detect basic body postures and study gait in PD patients [5]. However, EMG does not directly measure movements while a large number of electrodes may be needed to study complex movements. Recent developments in microelectronics have led to design and production of a new generation of small, cheap and robust sensors that can be used to measure kinematic parameters of the movements of the body segments. These developments have breathed a new life in the design of ambulatory systems for long-term monitoring of body movements. Accelerometers and gyroscopes have been used to detect and quantify tremor [6–10], bradykinesia [6,11] and LID [6,12–16] in PD patients. Ambulatory gait analysis systems [17] have been designed based on accelerometers [18–20] and gyroscopes [20,21] for healthy subjects, elderly and pathological cases. These sensors have been used as activity monitor [22] or for the classification of different body postures [23,24].

The market offers plenty of solutions concerning the remote health monitoring of people with pathologies involving the motor system such as PD [25,26]. Some examples of products and projects include (all products and projects are summarized in the Discussion section):

- Physilog (Gait Up is the company that now commercializing the Physilog [27]) is an ambulatory system for body motion analysis [7,21–24,28]. Movements are measured in one or three dimensions using one or multiple kinematic sensors. Single and three axis sensors are fixed on the body through a belt or directly attached on the skin using medical tape and supports. The system assesses motor function in Parkinson's disease, and functional disabilities during daily activities of the patients. Combination of the signals from different sensors is taken into account to address Tremor, Akinesia and Dyskinesia.
- Portable Motus System [29] quantifies movement bradykinesia, dyskinesia and tremor, monitors efficacy of drug treatment for movement disorders using a unique miniature solid state gyroscope, which however senses only rotational motion.
- The Kinesia [30,31] system integrates the measurement of electrical muscle activity (EMG) and motion using microelectrical mechanical accelerometers and gyroscopes. The device is worn on the wrist and finger while user interface videos guide the patient through upper extremity exercises normally completed in the clinic. Data is wirelessly transmitted to a computer for display, processing and storage. Potential applications include continuous remote monitoring of symptom fluctuations, better tuning of treatment interventions and quantifying motor effects during pharmaceutical development.
- APDM [32] produces a suite of wearable inertial measurement units and a system for assessing gait and balance called Mobility Lab. APDM's Mobility Lab is a portable gait and balance laboratory made for clinicians and therapists. It was designed to streamline gait and balance assessments, by making it easy to collect, store, and analyze data involving human subjects and APDM's wireless Opal movement monitor. Mobility Lab provides an intuitive graphical user interface for assist administrators to conduct a study. The administrator can repeat instructions, abort and repeat trials as necessary, and receive a clear indication of when the data collection is completed and all necessary data has been collected.
- Lift Labs [33] has developed three products a smart spoon (called Liftware Spoon) and two software applications (called Lift Pulse and Lift Stride) in order to help patients who suffer from PD and essential tremor.

However, most of these commercial products have limited focus on a single motor symptom and not overall assessment and lack important characteristics for Parkinson's disease and monitoring services such as: long-term recording, qualitative and quantitative assessments, high reliability, sensitivity and specificity. Also, a few projects have been funded, most of them covering the assessment of motor performance and the design of training and rehabilitation programs for PD patients, by incorporating virtual reality auditory feedback, interactive video conference technologies and conventional kinematic analysis:

- The Rescue Project [34] investigates a physiotherapy technique to improve mobility for people with Parkinson's disease. It introduces a rehabilitation program based around the concept of cueing and focuses mainly on the effects of bradykinesia and akinesia on walking and everyday activity. The cueing techniques used can improve the quality of walking and gait-related activities by providing an alternative means to guide movements, helping in overcoming and preventing so-called freezing episodes in which patients with PD report “being glued to the ground”.
- PARREHA (PARkinsonian REHAbilitation) [35] assistive technology in Parkinson's rehabilitation is a project concerned with the assessment of motor performance and design of “therapeutic” Virtual reality exercises supervised by video-conferencing. ParkService extended the PARREHA support to sources of exclusion not specific to PD by providing wireless home connectivity to specialized carers and also off-line services such as reminders to the user of their personal drug regime. ParkAid [36] also developed ParkWalker—concentrating on ease-of-use and comfort. ParkWalker, which recently took the commercial name INDIGO (INDependent I GO), is based upon a small display which clips on to a normal pair of glasses. Visual stimulation is generated by a small portable device which can also wirelessly connect to a clinician when the user is at home.
- DAPHNE (Detection of Activity Performance for Health with New Equipment) [37] is another project, allowing for quantitative measurement of neurological and psycho-physical health state. It proposes a portable computerized instrument that measures fundamental parameters of Parkinsonian reactive capabilities. The patient can perform several tasks like button pressing and vocal feedback in response to acoustic and visual stimuli. The system collects all reactive parameter changes, caused by different reasons such as stress or fatigue, and transmits them to operational health centers so that a wellness rate can be obtained.
- The HELP (Home-based Empowered Living for Parkinson's Disease) [38] intends to provide Parkinson Patients with a system that can supply specific amounts of drug according to their physical activity requirements at any moment. The HELP system is made up of a wearable subcutaneous pump, an intraoral cartridge inserted in patients' mouth, a wearable movement sensor, a blood pressure device and a control system that is constantly sending data, checking the patient and calculating the right quantity of drug to be supplied.
- CuPiD (Closed-loop system for personalized and at-home rehabilitation of people with Parkinson's Disease) [39] provides personalized rehabilitation exercises for people with PD at home. CuPiD aims to develop and test a combination of services for at home rehabilitation and training of major motor impairments caused by PD.
- REMPARK (Personal Health Device for the Remote and Autonomous Management of Parkinson's Disease) [40] develops a Personal Health System, featuring closed-loop detection, with response and treatment capabilities, for the improved management of PD patients. The REMPARK system is composed of two elements. The first element is a bracelet equipped with a sensor for measuring tremor in patients, and an inertial system worn at the waist on a belt made of biocompatible material. The second part, having the size of a mobile phone, is equipped with sensors and can process and wirelessly transmit the data collected.

**Figure 1.**
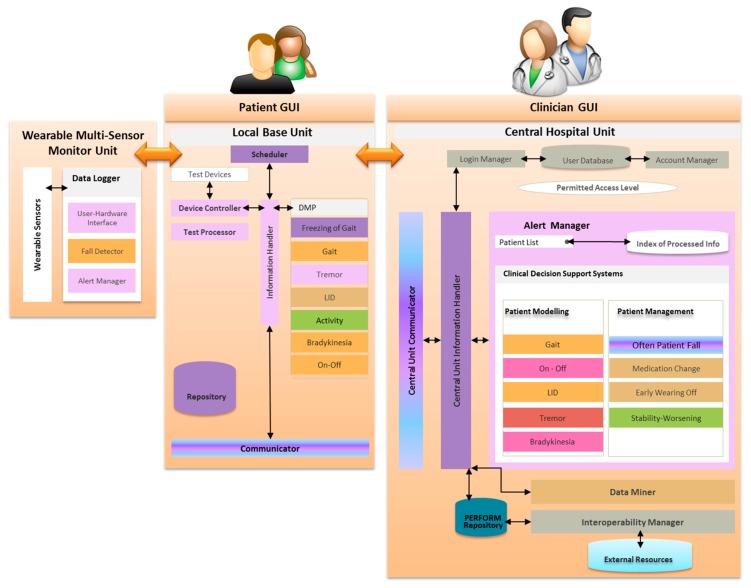
The PERFORM system architecture.

In this paper, the PERFORM system is presented (Figure 1). The system is designed and implemented to tackle problems associated with the efficient remote health status monitoring, the qualitative and quantitative assessment and the treatment personalization for people suffering from PD. The system is based on wearable accelerometers and gyroscopes for monitoring the disease evolution and intelligent techniques for detection and assessment of common PD motor disabilities. The system has been evaluated under real clinical conditions. A difficulty that physicians have to deal with is the inefficient and non-objective recording of the disease symptoms like tremor, levodopa-induced dyskinesia (LID), bradykinesia, freezing of gait (FoG) and falling. The daily symptoms and times of crisis are not adequately described by the patients and, on the other hand, a short office or hospital visit or medical examinations cannot provide a clear picture of the patient's status and the disease progress. The PERFORM system becomes the mediator between the physician and the patient by collecting all necessary information on a daily basis allowing thus the physician to be constantly informed about the patient's clinical state and readjust appropriately the treatment plan by changing the medication dosage and food intake.

## 3. The PERFORM System

The PERFORM system consists of three subsystems: the Wearable Multi-Sensor Monitor Unit, the Local Base Unit and the Centralized Hospital Unit (Figure 1).

### 3.1. System Architecture

#### 3.1.1. Wearable Multi-Sensor Monitor Unit

**Figure 2.**
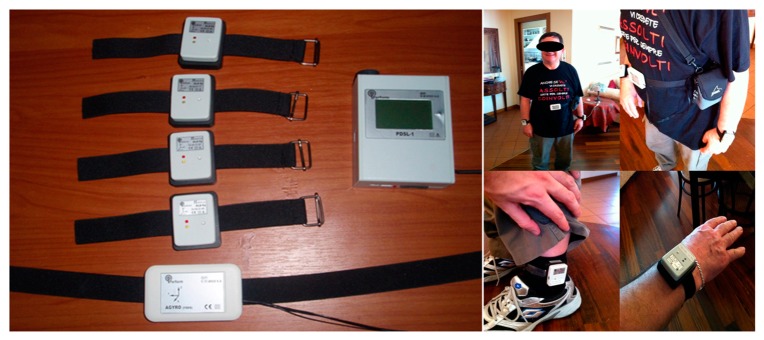
PERFORM wearable multi-sensor monitor unit (WMSMU) (**left**): four ALA-6g accelerometers, one AGYRO device (accelerometer/gyroscope) and the Parkinson Daily Data Set Logger (PDSL)-1 device. PERFORM WMSMU placement on the body of a Parkinson's disease (PD) patient (**right**).

The PERFORM wearable multi-sensor monitor unit (WMSMU) is physically attached to PD patient's body. The key role of this unit is to facilitate the monitoring of patient's daily motor activity and status through the continuous recording of specific signals. WMSMU is a light-weight wearable device composed of four tri-axial accelerometers (ALA-6g accelerometers, (ANCO S.A., Athens, Greece)) used to record the accelerations of the movements at each patient extremity, one accelerometer/gyroscope on the waist (AGYRO device (ANCO S.A., Athens, Greece)) used to record body movement accelerations and angular body velocity during body turning, and one data acquisition unit which is called Parkinson Daily Data Set Logger (PDSL-1 logger, (ANCO S.A., Athens, Greece)), receiving all recorded signals. The AGYRO device must be permanently attached to the PDSL-1 device by means of a long wire, while the ALA-6g accelerometers communicate wirelessly making up a body sensor network (Figure 2).

The sensors position was chosen after a detailed comparative study, with different sensors' locations and combinations. The outcome of the study (*i.e.*, sensors' locations and combination) has been used in the PERFORM system. The signals recorded through the five sensors are transferred to the Local Base Unit (see details in Section 3.1.2) where they are stored and processed.

##### WMSMU Functionality

The PDSL-1 logger supports two modes of operation: (i) Personal Computer (PC)-Connected Mode; and (ii) Normal Operating Mode (stand-alone).

**Figure 3.**
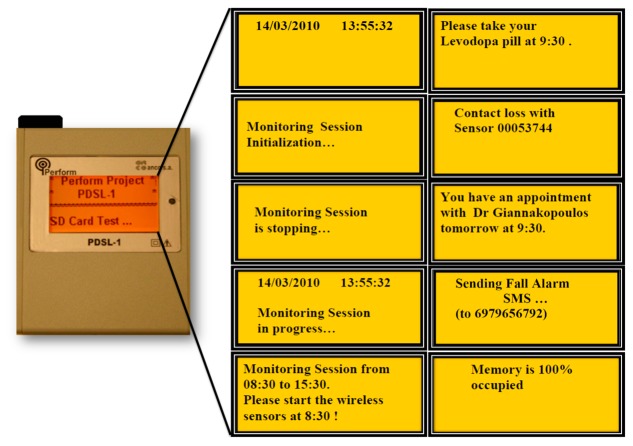
PDSL-1 logger (Normal Operating Mode): messages and alerts.

(i) *PC-Connected Mode*. The PDSL-1 logger enters automatically to this mode of operation, when a connection with a PC via a standard miniB USB cable is detected during power-on. In PC-Connected Mode the user can perform the following tasks by means of the Simple Detector software tool:
  - Get/set the device date and time.
  - Configure the device operating parameters.
  - Design the Monitoring and Testing schedules.
  - Acquire the Monitoring and Testing sessions sensor data stored into the SD card.
  - Design the patient Medicine and Appointment schedules.
  - Acquire events occurred.
  - Acquire device malfunctions.
  - Wireless sensor's contact loss/re-connection.
(ii) *Normal Operating Mode (stand-alone)*. The PDSL-1 logger enters into this mode of operation, when a USB connection is not detected at power-on (Figure 3). In Normal Operating Mode, the PDSL-1 logger conducts the following tasks:
  - Executes the predefined Monitoring Schedules.
  - Executes the predefined Testing Schedules.
  - Stores sensor data into the SD card.
  - Alerts the patient about the medicines according to the predefined Medicine Schedules.
  - Alerts the patient about the appointments with the doctors according to the predefined Appointment Schedules.
  - Sends SMS in the case the patient presses the Emergency button or when a patient fall is detected.
  - Receives Acknowledged SMS.
  - Detects sensors contact losses and reestablishments.
  - Monitors memory occupation.

#### 3.1.2. Local Base Unit

The Local Base Unit (LBU) is composed of a touch screen computer which is located in the patient's setting along with the WMSMU and the test devices. It is mainly responsible for the downloading, storage and processing of the raw signals coming from the test devices and the WMSMU, the identification and quantification of motor symptoms, the UPDRS evaluation of the patient and the patient's diary keeping (entries of time of drug and food intake). The signals coming from the PERFORM WMSMU are processed by the Daily Monitoring Processor (DMP) which is composed of the following modules: Tremor (Posture and Resting) Recognizer, LID Recognizer, FoG Recognizers, Bradykinesia Recognizer and Activity Recognizer. Furthermore, additional data coming from the test devices (glove, camera and microphone) are obtained through the Device Controller and are processed by the Test Processor, which is the component of LBU, responsible for controlling the functionality of test devices. The Test Processor acts as an intermediate between the Test Devices and the LBU, implementing operations concerning the calibration of the test devices, the management of the connection established between the LBU and the test devices, as well as the data handling of the content recorded by the device. Finally, the Test Processor provides the functionality for performing patient facial expressions and voice recordings, using the camera and the microphone, and recording tests (e.g., Hand Moving, Alternate Hand Moving, Fist/Open Close, *etc.*) with the glove device. All recorded data are played back/viewed by the medical doctor in order to have a better assessment of the patient's motor status.

There are several other modules in the LBU subsystem; each of them is playing a distinctive role. The Scheduler is in charge of monitoring the different patient schedules (monitoring, testing, medication, appointments) and providing reminders to the patient through the LBU user interfaces. It is also responsible for the synchronisation of old and new schedules (updated by the clinician in the Centralized Hospital Unit) and the transfer of these schedules to the wearable multi-sensor monitor unit. The Information Handler controls all the processes and workflows executed and provide a data access layer to the LBU Repository. All modules in the LBU are interfacing with the Information Handler and their outputs are transformed internally in order to be used as inputs for other modules. Finally, the Communicator is responsible to retrieve the latest data from the LBU, compose XML messages, encrypt them and transfer them to the Centralized Hospital Unit.

##### LBU Functionality

**Figure 4.**
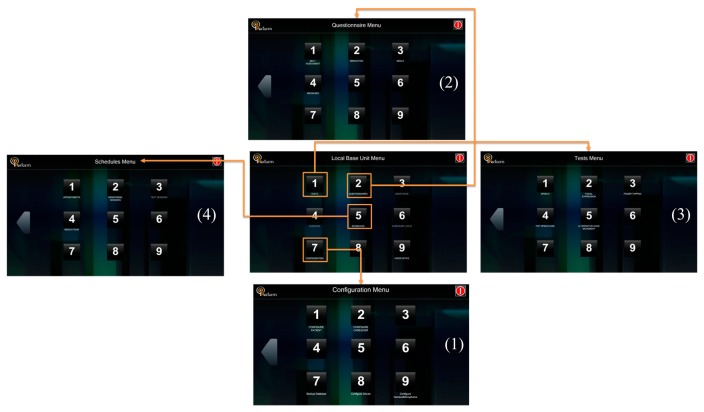
The main screen of P-GUI (Local Base Unit) menu is split into the following tasks: (1) Configuration Menu (touch button); (2) Questionnaire Menu (touch button); (3) Tests Menu (touch button); and (4) Schedules Menu (touch button).

The Patient Graphical User Interface (P-GUI) of LBU supports different modes of operation (Figure 4). Emphasis is given in designing an easy to use interface for the PD patient, considering the patient motor disabilities and limited computer familiarity. The designed interface is based on a touch-screen menu, and all system choices are based on it. The same options are given to the caregiver in case the patient is not capable of using the system due to the severity of his/her symptoms. The PD patient uses the interface to declare their subjective estimation of their own status, to gain access to relevant disease information, to receive instructions on life-style interventions, such as medication and food intake and on the execution of tests. Moreover, PD's patients declare medication intake information, which is useful for the patient status assessment. The main functionalities of P-GUI include: (i) Changing Personal Data; (ii) Checking Appointments Schedule and Monitoring Sessions Set-Ups; (iii) Inserting Medication, Food Intake Information and filling out a Self-Assessment Questionnaire; and (iv) Performing Test Sessions.

**Figure 5.**
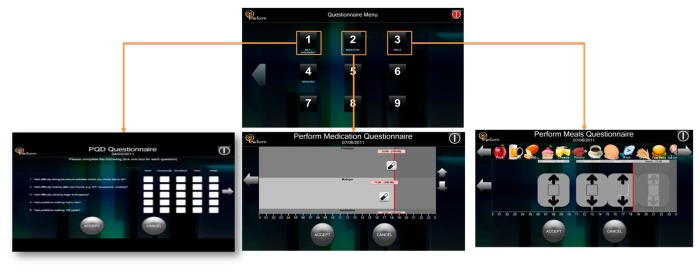
P-GUI Questionnaire Menu screen: Self-Assessment Questionnaire (**left**); Medication intake information (**middle**) and Food intake information (**right**).

**Figure 6.**
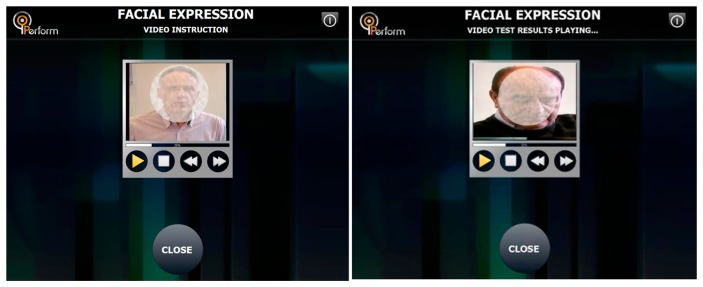
Instructions to patient screen (**left**); Patient's facial expression recording screen (**right**).

(i) *Changing Personal Data*. The patient data are shown in the P-GUI. The personal information can be changed according to the patient's inputs or doctor's suggestions.
(ii) *Checking Appointment Schedules and Monitoring Session Set-Ups*. The appointment and monitoring sessions are shown daily in the P-GUI. Once the doctor schedule a new appointment or monitoring session, the patient can check it in the Schedules Menu, respectively.
(iii) *Inserting Medication, Food Intake Information and Filling out a Self-Assessment Questionnaire*. Through the Questionnaire Menu screen (Figure 5), the patients can insert information regarding medication intake (kind, dose and time) and meals (type of food, amount, time). Also, they can access and fulfill the auto-evaluation questionnaire (Parkinson's Disease Questionnaire 39-PDQ39) for the quality of life).
  It is important to correlate this information with motor fluctuations and dyskinesia: motor behavior strongly depends on the assumption of the medication (in the usual patient's dosage) and the extent by which the metabolism of the drug is influenced by the diet (proteins or fats).
(iv) *Performing Test Sessions*. Through Tests Menu screen, the patient is able to initiate any of the available tests such as Speech, Face Expression, Finger Tapping, Fist Open/Close, and Alternative Hand Movement using the virtual reality glove (standalone application) as well as the microphone and video camera of the touch screen. The patient performs the tests as instructed by the visual interface of the P-GUI (Figure 6).

#### 3.1.3. Centralized Hospital Unit

The Centralized Hospital Unit (CHU) is positioned to the clinician's setting. The CHU is dedicated to processing all patient data and assisting the treating clinician in making appropriate treatment decisions. The CHU subsystem is responsible to further process the classified results of LBU in order to extract further knowledge and to generate alerts to inform the clinician for the patient condition. The three core components of the CHU subsystem are: (i) the Alert Manager; (ii) the Information Manager; and (iii) the Interoperability Manager.

The Alert Manager is the administrator of the Clinical Decision Support Systems (CDSS). It is the only module at the CHU subsystem which is aware of the internal data flow and data dependencies of the various modules in the CDSS, so its role is crucial for the system operation. Depending on the data arriving from the numerous LBUs, the Alert Manager creates the workflow that needs to be executed and then triggers all relevant submodules or CHU models/manager (Gait, ON/OFF, LID, Tremor, Bradykinesia, Often Patient/Fall, Early Wearing Off, Medication Change Proposer and Stability/Worsening). Depending on the alerts generated by these submodules, the Alert Manager handles the prioritization and representation of the newly produced alerts. During this process, the Alert Manager is responsible to keep a log, that contains information about the start and end time of each sub module's process for a specific patient, so as to check if every operation runs on time and the overall system's performance.

**Figure 7.**
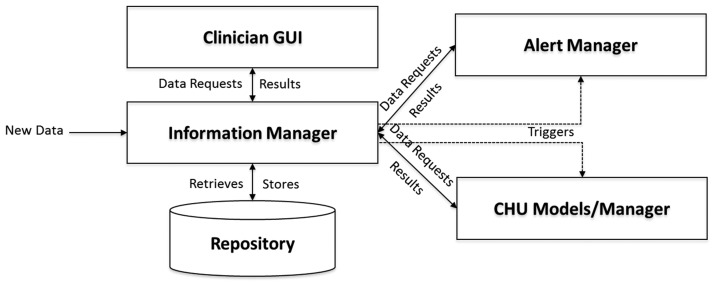
Data flow in the Perform system.

The Information Manager is responsible of fulfilling the needs for data retrieval and storage of the rest of CHU modules. It triggers the execution of other modules, depending on the information received, and stores the produced data into the central unit Repository. All PERFORM submodules interact with the information manager and cannot interact with the PERFORM repository (Figure 7). Also, the Information Manager is responsible for handling the LBU's User Data Requests, generated by the user interface.

The Interoperability manager is the link between the PERFORM system and external hospital and clinical information systems. It coordinates the overall information exchange between these legacy systems and through the information handler it may provide a more holistic view of the patient status, providing past information stored in external systems. All functions of the information manager are utilized by the PERFORM clinicians, yet they are transparent to these end users.

##### CHU Functionality

The Clinician Graphical User Interface (C-GUI) is a web-based application, which can be accessed either locally or remotely by the treating clinician and the general practitioner, using either a large or small screen access device (e.g., PC, laptop). Clinicians are directed to the home system screen, which presents the produced patient alerts to the patient specific screen, which provides the information needed to evaluate visually the patient condition. On request, the actual recorded signal and tests are downloaded from the patient-side to the healthcare center for review. The focus is on the provision of an adequate visual description of the patient status within one screen, minimizing the time spend by a clinician. Clinicians access the system periodically to check patient status, but the option to be alerted when the patient's status changes is also available.

The key functionalities of C-GUI include: (i) Searching for a Specific Patient; (ii) Extracting Patient Summary; (iii) Performing Patients Tests; (iv) Extracting Information on Symptoms Appearance; (v) Extracting Information about ON/OFF Periods; (vi) Changing medication intake; (vii) Changing an appointment.

**Figure 8.**
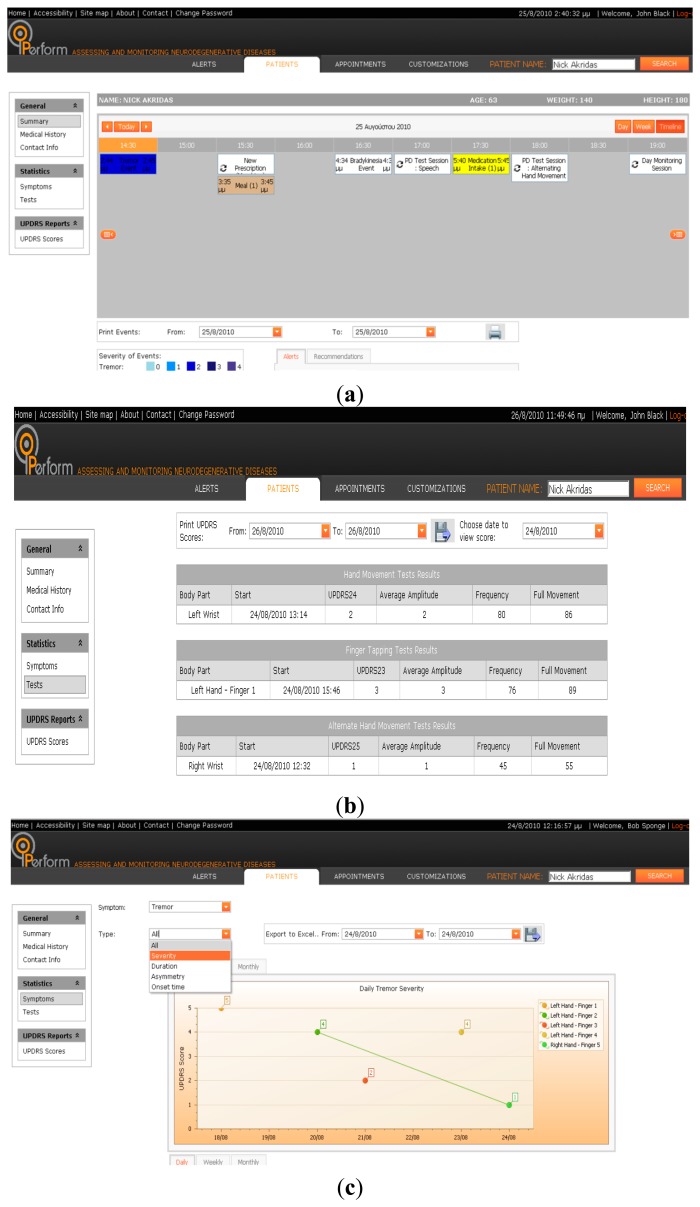

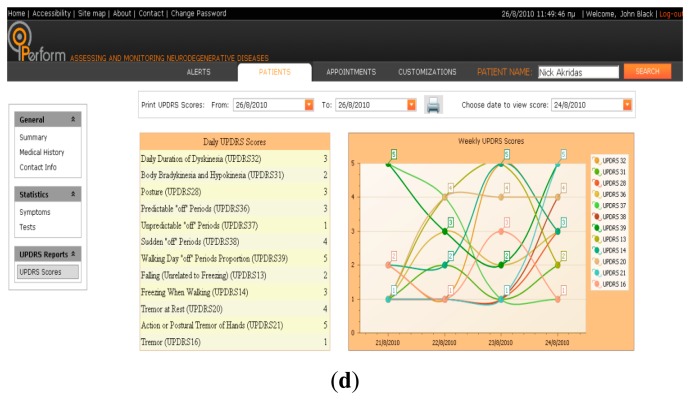
C-GUI functionalities (**a**) patient summary report; (**b**) tests results page; (**c**) symptoms page; (**d**) on-off results page.

(i) *Searching for A Specific Patient*. This functionality allows the clinician to search for a specific patient in the system's database
(ii) *Extracting Patient Summary*. An overview of all patient events is presented on a page (Figure 8a). Tremor, LID, Bradykinesia, Freezing Events, Falls, Test results, Meals and medication intakes are presented (depending on the time each one happened) on a scheduler timeline. In addition, a clinician is able to propose a new medication to the patient or ask the patient to run several tests. These test results will also be shown on the scheduler, as soon as is defined by the treating clinician.
(iii) *Performing Patient Tests*. Each patient performs tests by using a monitoring set. All the results of these tests are available to the clinician on a Test Page (Figure 8b).
(iv) *Extracting Information on Symptoms Appearance*. Tremor, LID, events and falls are the four symptoms which are represented employing graphs (Figure 8c). For each of the above symptoms, one daily, one weekly and one monthly graph is created for the duration (Tremor, LID and Bradykinesia), the severity (Tremor, LID, Bradykinesia), the asymmetry (Tremor, Bradykinesia), the onset (Tremor, LID, Bradykinesia) and for the number of falls the patient had which were or not connected to freezing. The clinician is able to choose the symptom he/she wants to view on graphs from the first drop-down list. Then he/she can choose to view all or one of the graphs separately, by making a selection from the second drop-down list.
(v) *Extracting Information about ON/OFF Periods*. The clinician can extract information about the ON/OFF period accessing a specific interface page (Figure 8d), which presents all UPDRS Scores for the selected patient.
(vi) *Changing Medication Intake*. The clinician can prescribe a new medication to the patient and ask to perform some tests or day monitoring sessions.
(vii) *Changing An Appointment*. Clinicians can define and review their appointments with their patients, using the scheduler, on which all appointments are depicted as boxes on the time line which corresponds to the time of the appointment. If the clinician has made his/her customizations concerning working hours, this scheduler (calendar) starts from the working hours of each clinician.

### 3.2. System Intelligent Modules

The core of the PERFORM system is a set of intelligent modules capable of automatically assessing PD common motor disabilities based on processing of the accelerometer and gyroscope signals and machine learning techniques. In order to provide a complete tool to the neurologist, the PERFORM system includes intelligent modules for tremor, bradykinesia, LID and FoG, which comprise the most common PD motor disabilities, along with several additional tools.

#### 3.2.1. Functionality

**Figure 9.**
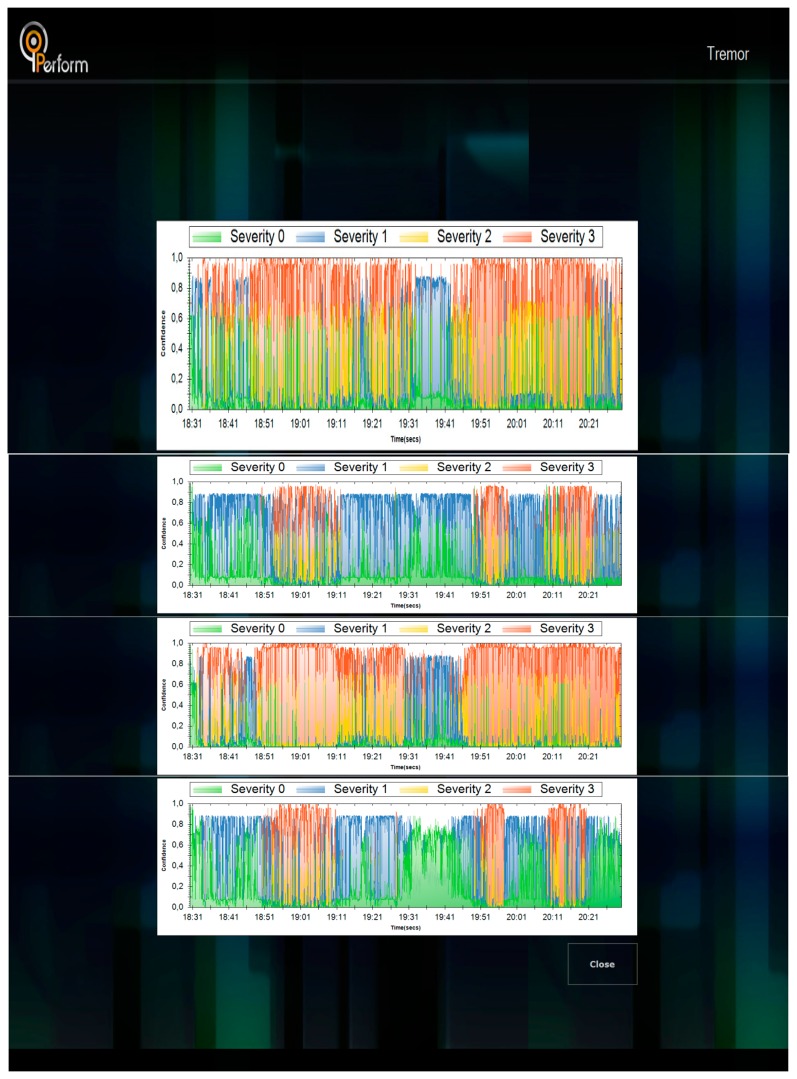
Results for Tremor analysis: (from top to bottom) left wrist, right wrist, left leg, right leg.

**Figure 10.**
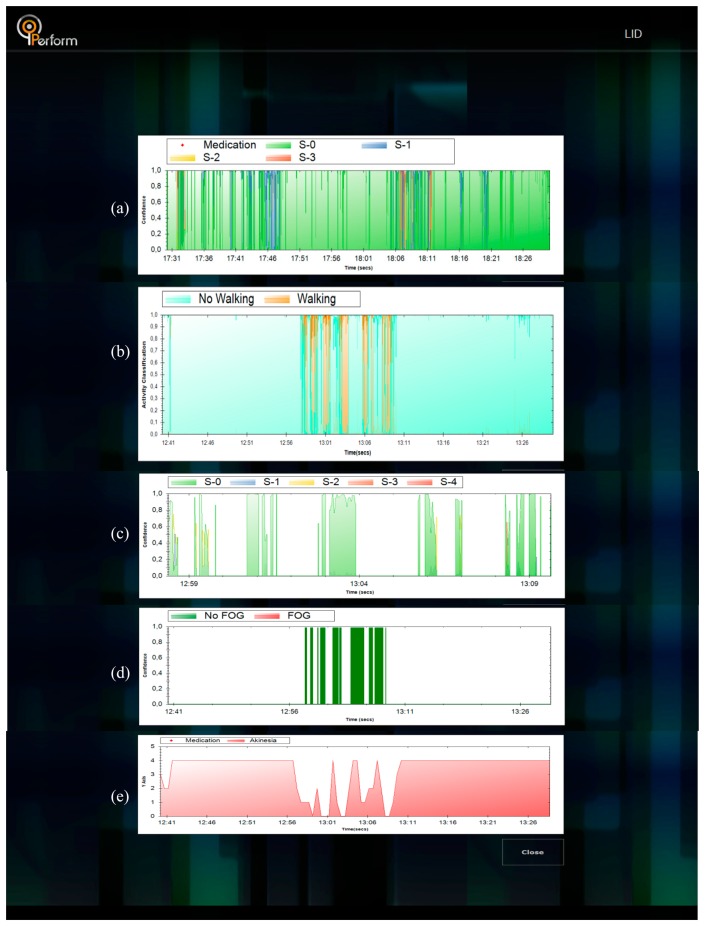
Indicative results for (**a**) Levodopa-induced dyskinesia (LID); (**b**) activity; (**c**) bradykinesia; (**d**) freezing of gait (FOG) and (**e**) akinesia (from top to bottom).

Upon connection of the data logger to the LBU, all files included in the data logger are identified and can be downloaded locally, *i.e.*, from the data logger to the LBU. Then the selected signal is downloaded and processed using the set of intelligent modules. After processing is finished, the results menu is displayed. By selection the appropriate action, the results of the analysis of the signal is displayed (Figure 9). In the same context, results can be presented for all functionalities presented in Figure 10.

The graphs represent the confidence of the decision for each severity (with different colors) with respect to time, *i.e.*, for the left wrist (top graph of Figure 9), from 19:31 to 19:41, the result with the highest confidence is severity 1 (with blue color), while from 19:41 to 20:21, the result with the highest confidence is severity 3 (with red color) with some periods with high confidence for severity 2. From the example it is clear to observe that this patient had a lot of tremor, however mainly located to his/hers left side (left wrist and left leg).

#### 3.2.2. Intelligent Modules Creation

All intelligent modules have been developed using a database of short-term (∼15 min) recordings, created using the recording device described above, in hospital environment. The recording started with the subject lying on the bed and the protocol consisted of three major tasks: (i) lying on the bed; (ii) rising from the bed and sitting on a chair located by the bed; and (iii) standing up from the chair and performing a series of tasks (walking, opening and closing a door, drinking, random movements). During the recording, the subjects were instructed to act freely, speak and make voluntary movements if they need to. The procedure (performing the tasks) was videotaped. Clinical annotation was provided by expert neurologists during the recording and afterwards via visual inspection of the video footage. For the clinical annotation, the UPDRS was used. The short-term dataset included 39 recordings from 24 patients in different states (ON/OFF) and included all motor symptoms. The short-term dataset is presented in Table 1. The methodological approaches used in each intelligent module (signal processing, window length, feature extraction and selection, classifier selection, classifiers parameters used) are based on previous studies [41–44], performed using subsets of the short-term dataset (except from [43], were a different dataset was employed). The intelligent modules were developed using the short-term dataset.

The PERFORM system was also used for long-term recordings. These included two recordings (∼4 h each, one in the morning and the second in the afternoon) for five consecutive days, at patient's home. The patient (or the caregiver) was asked to record his/her motor condition in the home diary every half an hour during the day, using a different level of classification for each PD symptom (e.g., ON-OFF state, bradykinesia, tremor or LID). Twelve patients were enrolled in the long-term recordings phase. The long-term dataset is presented in Table 2.

##### (1) Tremor Assessment Module

Tremor assessment module is based on the analysis of signals obtained from accelerometers attached to specific body segments [41]. Several features are extracted from the recorded signals, related to time and frequency domain characteristics, including features indicative of low frequency movements, which can discriminate tremor from the other PD motor symptoms. The features were extracted using a moving window with 3 s duration and 1.5 s overlap. Using a feature selection method [41], a subset of features is selected and incorporated into a Hidden Markov Model (HMM) for tremor severity recognition. For the discrimination of tremor type (resting/postural), spatial features were extracted based on the gravity force applied on each accelerometer axis and the angles between different body segments. Again, a subset of features is selected using a feature selection method, and these features are fed into a second HMM for body action-posture recognition. In both cases, feature selection was based on a Wrapper method [45], which makes use of the best-first search algorithm [46]. The information from the two HMMs is merged resulting into a thorough tremor assessment addressing both its severity and type. The numbers of instances per class label for the long-term and short-term datasets are presented in Tables 1 and 2, respectively.

##### (2) LID Assessment Module

The LID assessment module is based on the analysis of the signals recorded from the sensors [42]. The signals are analyzed using a sliding window of 1 s length and 0.5 s overlap, and several features are extracted (from each window), including mean value, standard deviation, entropy, energy in specific frequency sub-bands and entropy of the frequency spectrum. The numbers of instances per class label for the long-term and short-term datasets are presented in Tables 1 and 2, respectively. Based on these features, a decision tree is created using the C4.5 decision tree induction algorithm, and it is used for LID detection and severity classification.

##### (3) Bradykinesia Assessment Module

The classification methodology used for the bradykinesia assessment module includes three steps [43]. Initially the signals from the sensors are filtered using a band-pass filter with cut off frequency of 1–3 Hz [47]. Then, several features are extracted from the filtered signals, including Approximate Entropy, Sample Entropy, root mean square value, cross correlation value and range value, using a 5 s duration sliding window, with 50% overlap. These features are used as input into an SVM classifier (the numbers of instances per class label for the long-term and short-term datasets are presented in Tables 1 and 2, respectively). Several different feature combinations are tested and the best performing was selected (including Approximate Entropy, cross correlation value and range value).

##### (4) FoG Detection Module

The FoG detection module methodology consists of three stages [44]. In the first stage preprocessing of the signals is performed and then the signals are analyzed using a sliding window of 1 s length and 0.5 s overlap. The entropy of the signal for each axis of each sensor is extracted; these values formulate a feature vector which is used for the classification of each second of the recorded signals as FoG or not, based on a Random Forest classifier, which is a collection of tree-structured classifiers. For the construction of each tree of the forest a subset of samples is selected from the dataset, using the bootstrap technique, while each tree is built to the maximum size without pruning. In our study the Random Forests consist of 10 trees. The numbers of instances per class label for the long-term and short-term datasets are presented in Tables 1 and 2, respectively.

## 4. System Evaluation

### 4.1. Intelligent Modules Evaluation

The intelligent modules were initially evaluated using the short-term dataset, based on the leave-one-patient-out cross validation technique [48]. All datasets are unbalanced since the normal class is significantly larger than all other classes. To address this issue the instances of the normal class were subsampled in the training datasets (selecting 20% of the available number of normal instances for all assessment modules, except from the FoG module, where 4% of the available number of No FoG instances were used), while the testing datasets remained unaffected. The obtained results in terms of classification accuracy and recall (average for all classes) are presented in Table 1. Also, the long-term recordings were used for evaluation. The recordings were used as inputs in the intelligent modules (trained with all instances of the short-term dataset, using the normal class subsampling) and the output results were compared against the diary entries. The obtained results in terms of mean absolute error are presented in Table 3.

### 4.2. Wearability Analysis

The wearability analysis [49] was performed to identify if the PERFORM WMSMU is acceptable by the end users (PD patients) and how the design could be improved. However, since the design of a wearable device is clearly a multi-criteria optimization problem, a tested methodology that could provide useful insights on the problematic issues and will enable the optimization of the design is adopted. The methodology selected for the wearability evaluation of PERFORM WMSMU was mostly based on the work of Knight *et al.* [50]. The research performed confirmed that the wearability analysis of a device is a multifaceted problem; the wearable devices affect the wearer in different ways and thus there are several effects that should be taken into account when assessing the wearability of the device. These effects are ranging from physiological effects due to the attachment of extra load to the human body to comfort related effects. Given that each wearable device is designed and manufactured in order to provide a sophisticated functionality and to be used in some usage scenarios is adding more dimensions to the problem.

According to Knight *et al.* [50], there are three main types of physical effects to be assessed in order to ascertain a device's wearability: (1) physiological; (2) biomechanical; (3) comfort. It should be noted however, that there are several factors that can influence the extent to which a wearable device might affect the wearer. Some of them are related to the device characteristics while others are more related to the characteristics of the wearers.

Based on the aforementioned method and factors, an evaluation questionnaire was developed and employed during short-term and long-term recordings. This questionnaire was composed of the following sections:

- Section 1: Personal information (age, sex, *etc.*)
- Section 2: Posture Analysis: this section was to be completed by an observer and includes all the material related to the Rapid Entire Body Assessment (REBA) score chart [51].
- Section 3: Energy Cost Analysis: this section was to be completed by the PD patient according to his perception of extra energy used in order to perform daily activities.
- Section 4: Pain/Discomfort Analysis: this section was to be completed by the patient according to his perception of pain or discomfort to specific body areas (using the body maps) [52].
- Section 5: Comfort Rating Scales: this section was to be completed by the patient in order to provide his/her thoughts and feelings regarding to several issues related to comfort like emotions, anxieties and harm caused.
- Section 6: Interview and complementary questions: this section included both some complementary questions that are related to allergic reactions, excess heat and sweat caused by the device and also enable the user to provide his/her own suggestions on how the system can be improved.

**Figure 11.**
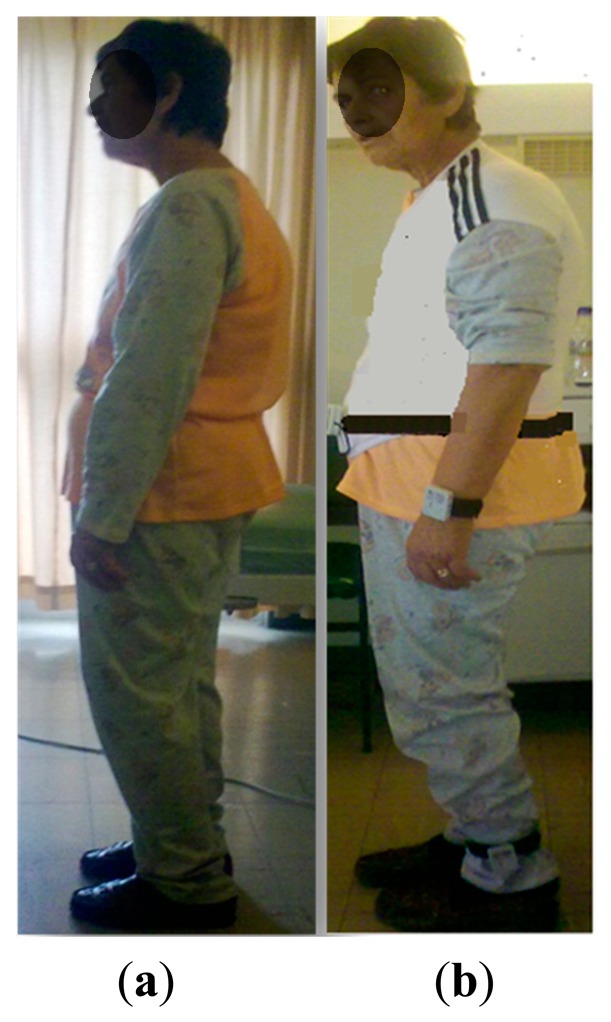
(**a**) A PD patient without wearing the PERFORM WMSMU; (**b**) A PD patient with PERFORM WMSMU. Posture Analysis was based on Rapid Entire Body Assessment (REBA) evaluation [49,51]. All patients were asked to stand up and stay still for a couple of minutes, while an observer completed the scoring cart of REBA evaluation based on the posture of trunk, neck, legs and arms.

The wearability analysis of the PERFORM WMSMU was completed with the involvement of 20 PD patients during short-term recordings and 24 patients during long-term recordings. From the analysis of results, two main conclusions were drawn:

- The PERFORM WMSMU does not change the posture of the patient and that it is not causing significant problems to the patients (Figure 11).
- The PERFORM WMSMU is considered wearable by all subjects (Table 4).

## 5. Discussion

The PERFORM system aims to tackle problems associated with the efficient remote health status monitoring, the qualitative and quantitative assessment and the treatment personalization for people suffering from neurodegenerative diseases and movement disorders, such as PD. The system is based on wearable monitoring devices (accelerometers and gyroscopes), which are wirelessly connected and seamlessly integrated to produce a user-friendly and patient-customized monitoring tool. The recorded signals are pre-processed and stored at the patient site. At the point of care (hospital center), the supervising health professionals are able to remotely monitor their patients, personalize their treatment and medication schedules and generate statistical data, so as to study and evaluate the efficacy of medication, based on the patients' specific personal and medical characteristics.

The PERFORM system aims to improve the prevalent philosophy in the follow up of PD patients and to change the paradigm in PD treatment. The new paradigm supports the continuous follow up of patients with easy to wear sensors and computer intelligence to be used by doctors in order to design optimized personalized treatment, delay the appearance of symptoms (such as LID) and improve quality of patients' lives. These are achieved through an assistive tool for assessing the course of the disease (in terms of motor disabilities) and evaluating the medication efficiency, through continuous monitoring and assessment of the disease evolution, automated diagnosis and decision support technologies. Disease motor symptoms are long-term monitored and quantified, significantly improving the current short-term subjective medical practice, allowing the health professionals to assess the disease's progress and evaluation/adaptation of the medical treatment based on the changing symptoms and efficiency of drug intake (type of drug, dose, timing).

Better control of the disease symptoms will have a synergistic positive effect on the psychosocial and physical functioning of the patients. Therefore patients are empowered to have their role in a society by living independently, working, participating in social activities and enjoying life. Although the final system has to be tested on many Parkinson's disease patients, bearing in mind the needs of other neurodegenerative diseases and Parkinsonian syndromes with similar motor and mental disabilities. Thus it offers a great potential for easy future use for most neurodegenerative syndromes (e.g., Huntington's disease, Tourette's disease, idiopathic dystonia).

PERFORM system targets cost and effort savings related to frequent visits to hospitals for assessment and treatment modification (widely used in current clinical practice), since it provides a monitoring tool for out-of-hospital long-term monitoring. Also, it provides work efficiency to healthcare professionals, through the utilization of multi-parametric information fusion, allowing all related information to be included in a single system.

In Table 5 a summary of existing commercial products *vs.* the PERFORM system is presented. Also, in Table 6 the competitive landscape of integrated projects for PD monitoring and management is presented. The current project implementations, developments, products and research activities are not sufficient for monitoring all the different parameters covering PD. The PERFORM system is based on a holistic approach, tackling all major PD motor disabilities and not focusing on only some of them. Also, it has been used for long-term recordings and the results indicate its high efficiency. Furthermore, it has been evaluated for usability and comfortless during the clinical trials. The outcomes of these trials conclude that the acceptance of this system was satisfactory. These clinical trials also provided useful insights and guidelines to lead to redesign of the system to improve patient compliance [49].

## 6. Conclusions

In contrast with other diseases there is no treatment and therapeutic schema for PD. The dosage and the way of medication administration are totally personalized for every patient. When the PD disease appears the treatment seems very simple but in the course of time the treatment becomes complicated and requires more and more the patient's participation. During the short visit of PD patient the clinician must be informed for the patient's day motor status. This is required to configure the treatment strategy, drug time intake, drug doses, intervals between doses, combination of drugs depending on the food intake and other details. The clinician tries to retrieve information for patient's motor status for the previous days or weeks. This is almost impossible since it is difficult for PD patients to describe their symptoms and they cannot assess exactly their reaction to the drug. Consequently, the clinician cannot receive proper information to define realistically the drug administration treatment. The PERFORM system offers daily assistance to the clinician neurologist who tries through conflicting information from the patients and their relatives to determine the optimal therapeutically schema. The PERFORM system is used by the PD patients and in a simple, safe, painless and non-invasive way to record patient motor status for long-time intervals. In this way, the clinician can have a precise, long-term and objective view of patient's motor status in relation to drug and food intake; all the aforementioned factors are directly involved in the drug absorption and action. With the PERFORM system the clinician can remotely receive precise information for the PD patient's motor status on previous days and define the optimal therapeutical treatment.

## Figures and Tables

**Table 1.** Short-term dataset, used for the development and initial evaluation of the Intelligent Modules.

**Motor Symptoms**		**OFF**										**ON**			
		
**Assessment Modules**		**Tremor**				**Bradykinesia**				**FoG**		**LID**			
				
**Status**		**Normal**	**Tremor 1**	**Tremor 2**	**Tremor 3/4**	**Normal**	**Brad 1**	**Brad 2**	**Brad 3/4**	**No FoG**	**FoG**	**Normal**	**LID 1**	**LID 2**	**LID 3/4**

**Total Duration of Recordings (h:min:s)**		06:41:22	01:04:50	00:20:18	00:12:09	07:38:04	00:21:48	00:11:10	00:04:57	08:11:40	00:07:00	06:11:48	00:59:05	00:53:26	00:14:21

**Number of Instances**		15,734	2,542	796	476	10,744	641	513	117	57,821	824	43,723	6,949	6,285	1,688

**Performance Metrics**	**Accuracy**	87%				74.5%				79%		85.4%			

	**Av. Recall**	84.8%				82.6%				80.6%		82.4%			

**Table 2.** Long-term dataset, used for the evaluation of the Intelligent Modules.

**Motor Symptoms**	**OFF**										**ON**			

**Assessment Modules**	**Tremor**				**Bradykinesia**				**FoG**		**LID**			

**Status**	**Normal**	**Tremor 1**	**Tremor 2**	**Tremor 3/4**	**Normal**	**Brad 1**	**Brad 2**	**Brad 3/4**	**No FoG**	**FoG**	**Normal**	**LID 1**	**LID 2**	**LID 3/4**
**Total Duration of Recordings (h:min:s)**	298:03:07	67:16:42	49:15:45	30:10:36	228:38:03	104:43:04	70:19:07	41:05:56	441:06:37	03:39:34	203:48:21	76:17:11	83:19:26	81:21:12
**Number of Instances**	354,086	79,927	58,524	35,850	162,971	74,643	50,123	29,295	1,572,118	13,042	726,365	271,885	296,967	289,944

**Table 3.** Summary of the results from all PERFORM Assessment/Detection Modules.

**PERFORM Assessment/Detection Modules**	**Dataset**	**Technique**	**Results (Classification Accuracy)**
Tremor	short-term dataset	HMM	87% classification accuracy
	long-term dataset		0.088 mean absolute error

LID	short-term dataset		85.4% classification accuracy
	long-term dataset	DT	0.31 mean absolute error

Bradykinesia	short-term dataset	SVM	74.5% classification accuracy
	long-term dataset		0.25 mean absolute error

FoG	short-term dataset	RF	79% classification accuracy
	long-term dataset		0.79 mean absolute error

**Table 4.** Average Level of effect of the PERFORM WMSMU to PD patients [49,50].

	**Metric**	**Units**	**Level of Effect per Patient**	
			**Average**	**Level of Effect**
**Energy cost**	Relative Perceived Exertion	Borg-CR score	0.71 */0.53 **	Low */Low **
**Biomechanical**	Posture	REBA score	0 */0 **	Low */Low **
	Localized pain/discomfort	Borg-CR score	0.1 */0 **	Low */Low **
**Comfort**	General Wearable	Average CRS score	3.16 */1.76 **	Low */Low **

* Evaluation from 20 PD patients during short-term recordings;

** Evaluation from 24 PD patients during long-term recordings.

**Table 5.** Systems for Parkinson's disease motor symptoms monitoring and analysis.

**System**	**Short Description**	**Addressable Issues**	**Commercial Product**
Physilog	Ambulatory system for body motion analysis Measures movements in one or three dimensions using one or multiple kinematic sensors	Tremor Akinesia Dyskinesia	No
Portable Motus System	Quantifies movements and motor symptoms, Monitors efficacy of drug treatment for movement disorders using a unique miniature solid state gyroscope	Bradykinesia Dyskinesia Tremor Dystonia	Yes
Kinesia	Captures motor symptoms at home Integrates the measurement of electrical muscle activity (EMG) and motion using microelectrical mechanical accelerometers and gyroscopes	Tremor Bradykinesia Dyskinesia	Yes
APDM	Includes a full suite of triaxial sensors Measures rotational rate, acceleration, magnetic field strength, and temperature	Usage for research and clinical purposes	Yes
PERFORM	Quantifies movements and motor symptoms Accelerometers and gyroscope sensors	Tremor, Dyskinesia Bradykinesia Freezing of Gait	

**Table 6.** Integrated Projects for Parkinson's disease motor symptoms monitoring and analysis.

**Project Acronym**	**Remote Monitoring**	**Feedback to PD Patient**	**Assessment**	**Address all Cardinal Motor PD Symptoms**	**Treatment Modification and Personalisation**	**Evaluation of New Drugs**
Rescue [34]	✓		✓			
PARREHA [35]	✓		✓			
DAPHNE [37]	✓		✓			
HELP [38]	✓		✓		✓	
CuPID [39]	✓	✓				
REMPARK [40]	✓	✓	✓		✓	
Proposed System	✓	✓	✓	✓	✓	✓
